# Supplementary figures and images for: Comprehensive analysis of full-length transcripts reveals novel splicing abnormalities and oncogenic transcripts in liver cancer
Source: PLoS Genet. 2022 Aug 4;18(8):e1010342. doi: 10.1371/journal.pgen.1010342 (PMC9380957; doi:10.1371/journal.pgen.1010342)

**S1 Fig.**

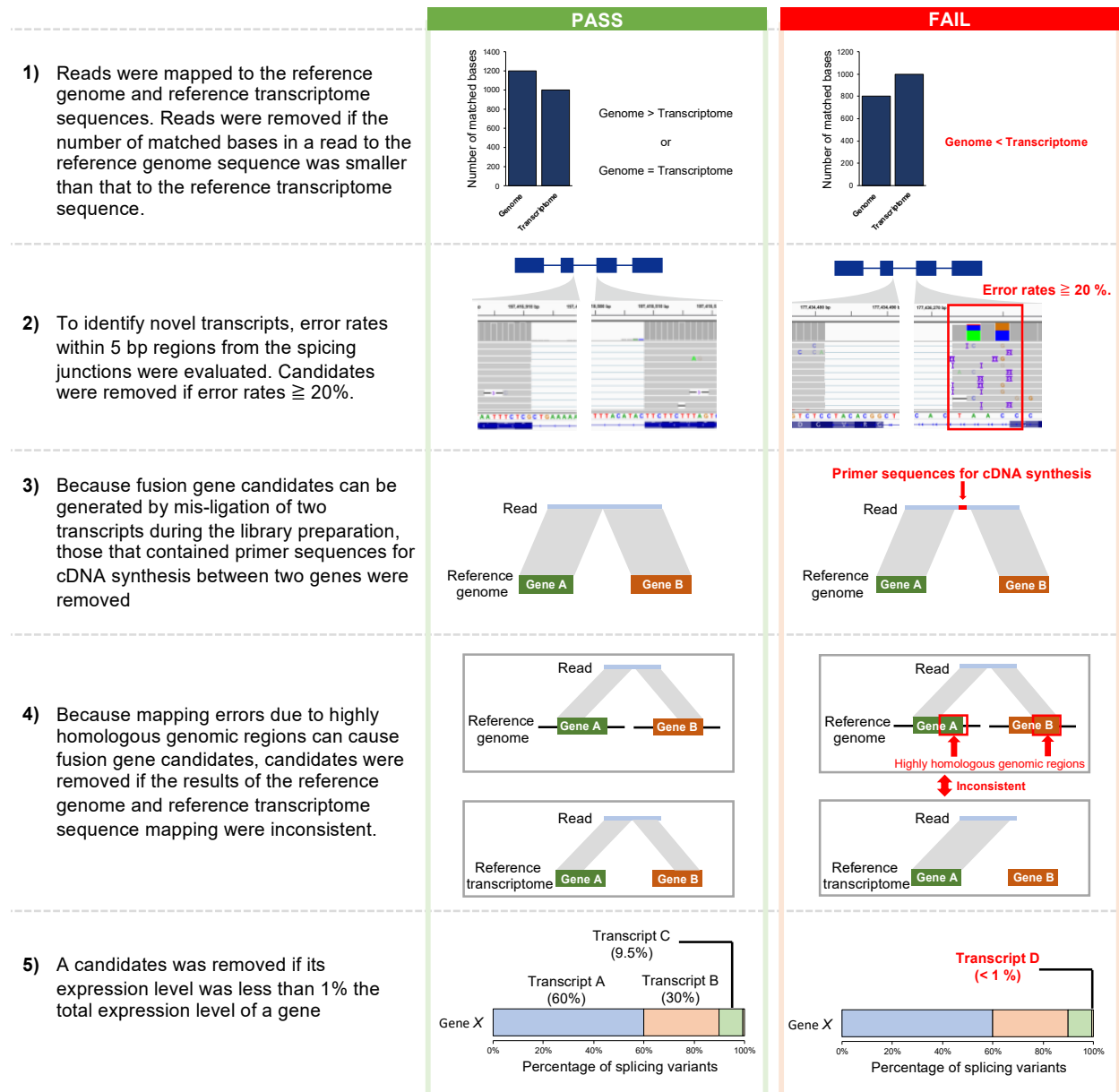

Supplement: S1 Fig — (PDF) [file pgen.1010342.s001.pdf]

**S2 Fig.**

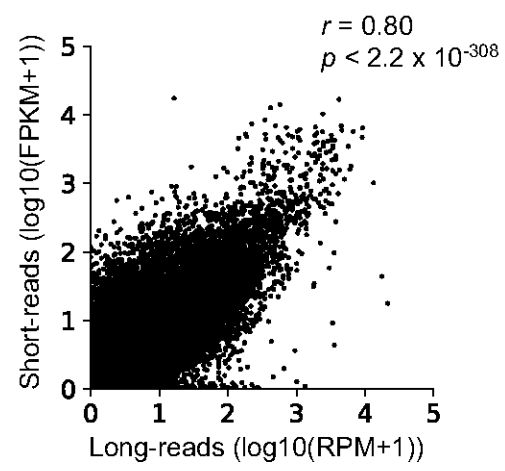

Supplement: S2 Fig — Transcript abundance was measured in reads per million mapped reads (RPM) for long-read RNA-seq data and fragments per kb of exon per million fragments mapped (FPKM) for short-reads RNA-seq data. log10 converted values of RPM+1 and FPKM+1 are shown. r, Pearson’s correlation coefficient; p, p-value. (PDF) [file pgen.1010342.s002.pdf]

S3 Fig.

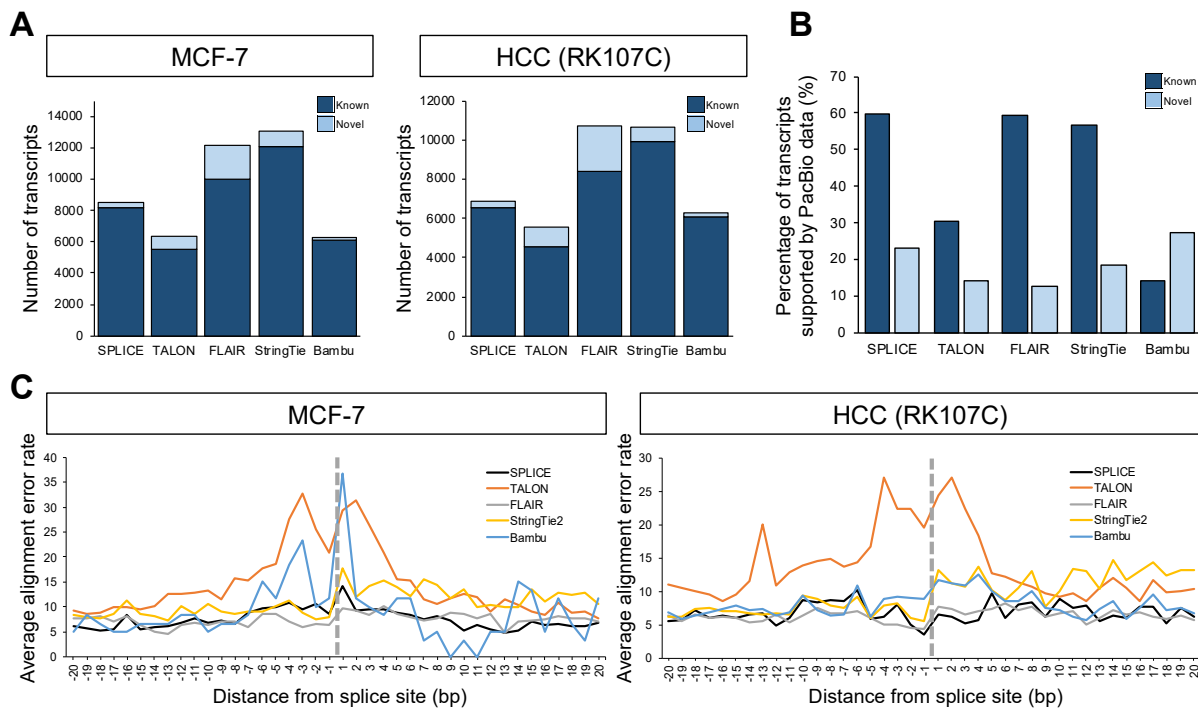

Supplement: S3 Fig — (A) Number of transcripts detected by SPLICE and four other methods (TALON, FLAIR, StringTie and Bambu). (B) Percentage of transcripts supported in PacBio data with SPLICE and the other methods. (C) Average alignment error rate of SPLICE and the other methods for novel slice sites. The gray dotted line indicates the location of the splice site. (PDF) [file pgen.1010342.s003.pdf]

S4 Fig.

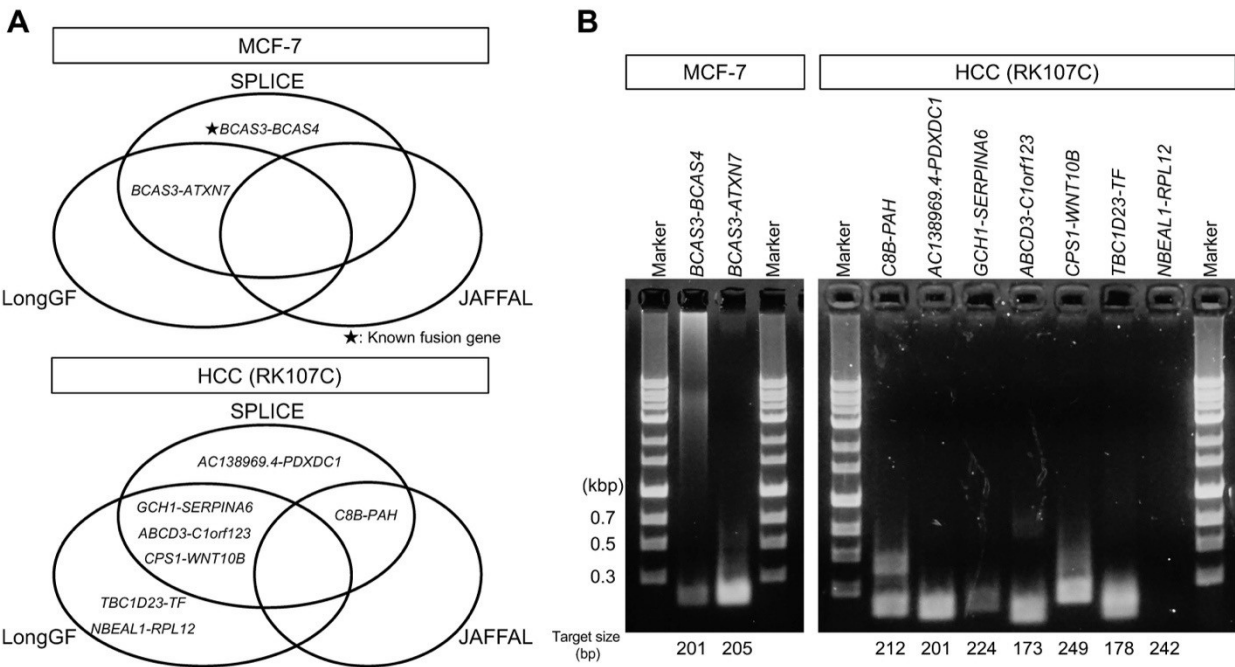

Supplement: S4 Fig — (A) Venn diagram of the fusion genes detected by SPLICE and two other methods (LongGF and JAFFAL). The neighboring gene pairs (< 200,000 bp) detected by LongGF (MCF-7: VMP1-RPS6KB1, GTF2IRD1-GFT2I and PITPNC1-BPTF, HCC (RK107C): C11orf52-DIXDC1) were excluded from the list because they cannot be distinguished from read-through transcripts without information on structural variations. (B) RT-PCR validation of fusion genes detected by SPLICE, LongGF, and JAFFAL. (PDF) [file pgen.1010342.s004.pdf]

S5 Fig.

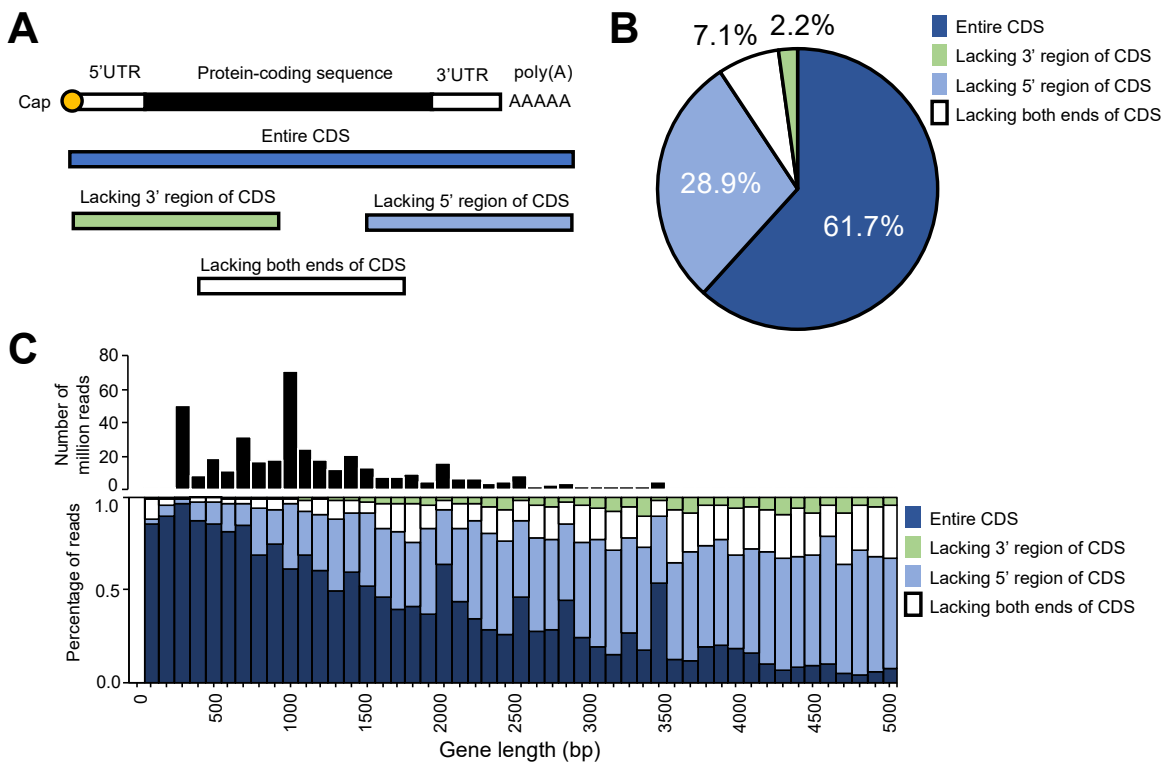

Supplement: S5 Fig — (A) Schematic diagram of the read length types. "Entire CDS" reads contain all coding exons of known transcripts. "Lacking 3’ region of CDS" reads lacks coding exons in the 5’ region of known transcripts. "Lacking 5’ region of CDS" reads lacks the coding exons in the 3’ region of known transcripts. "Lacking both ends of CDS" reads lacks both the coding exon in the 5’ region and the 3’ region of known transcripts. (B) Pie chart of the detected transcripts by read length type. (C) Relationship between the reference gene length and percentage of read length type in the mapped reads. (Upper panel) Distribution of mapped reads to the reference gene length. (lower panel) Percentage of the mapped read length type to the reference gene length. (PDF) [file pgen.1010342.s005.pdf]

**S6 Fig.**

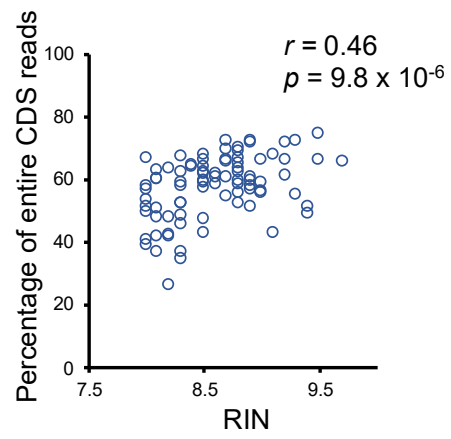

Supplement: S6 Fig — r, Pearson’s correlation coefficient; p, p-value. (PDF) [file pgen.1010342.s006.pdf]

**S7 Fig.**

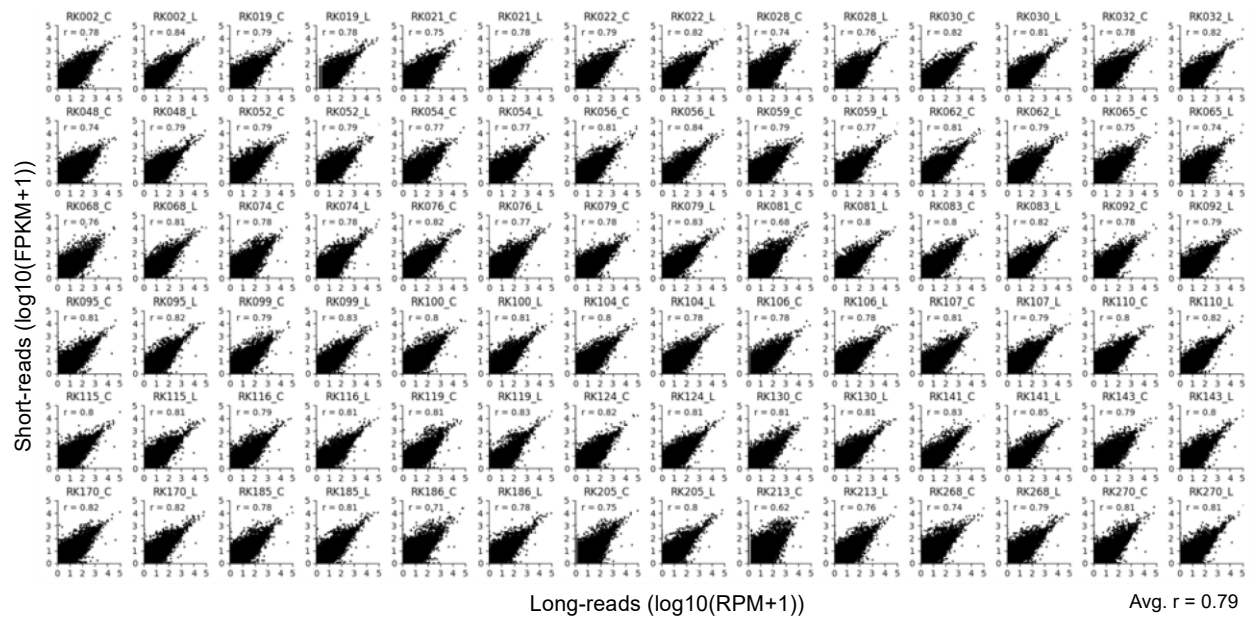

Supplement: S7 Fig — Transcript abundance was measured in RPM and FPKM for long-reads and short-reads RNA-seq data. log10 converted values for RPM+1 and FPKM+1 are shown. r, Pearson’s correlation coefficient. p < 2.2 × 10–308 for all samples. (PDF) [file pgen.1010342.s007.pdf]

S8 Fig.

A

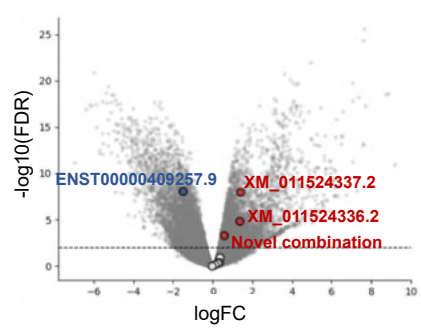

B

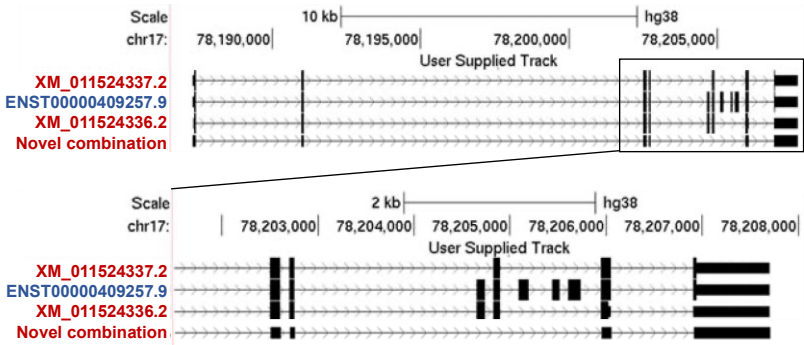

Supplement: S8 Fig — (A) Significantly up- and down- regulated AFMID transcripts in HCCs are shown in red and blue, respectively. (B) Structure of AFMID transcripts. (PDF) [file pgen.1010342.s008.pdf]

S9 Fig.

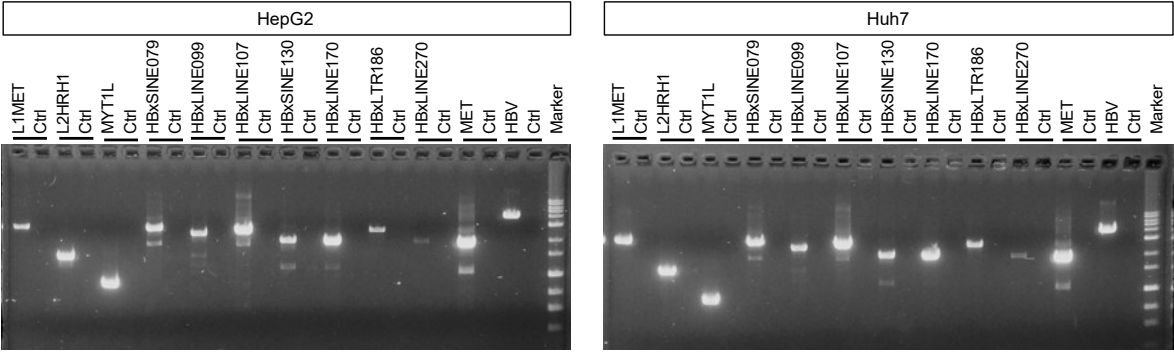

Supplement: S9 Fig — Target transcript size bands were confirmed by agarose gel electrophoresis. Cell lines transfected with empty vectors were used as control (Ctrl). (PDF) [file pgen.1010342.s009.pdf]

S10 Fig.

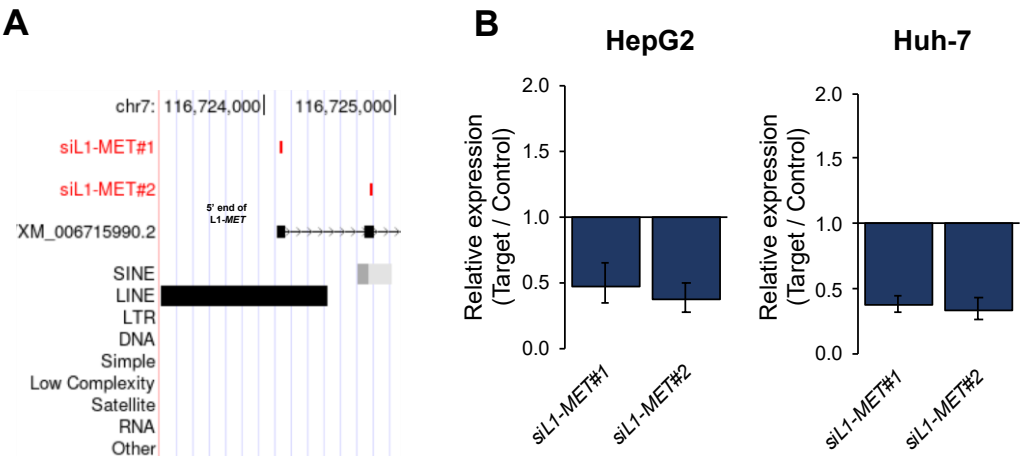

Supplement: S10 Fig — (A) Schematic diagram of the target sequence for L1-MET-specific knockdown. (B) Expression levels of L1-MET in L1-MET knockdown in both cell lines. (PDF) [file pgen.1010342.s010.pdf]

S11 Fig.

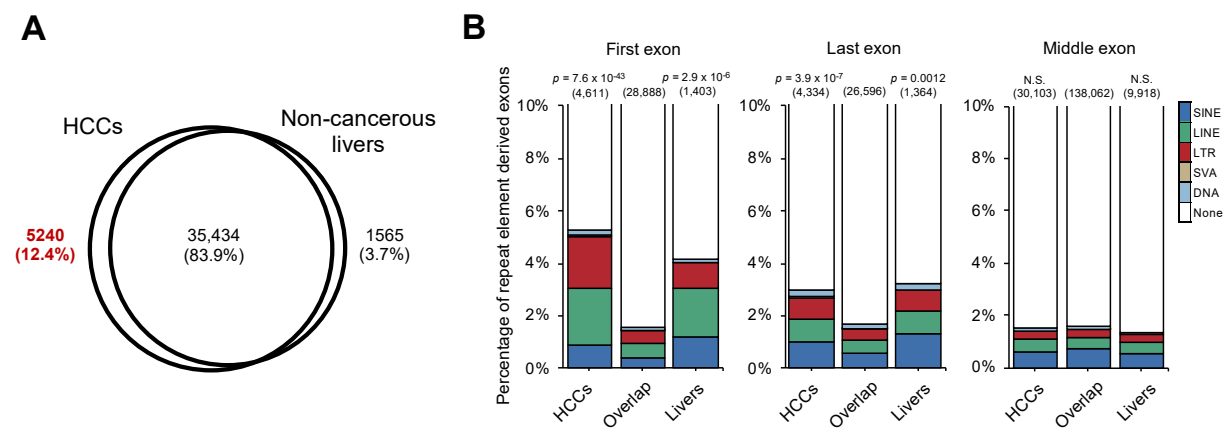

Supplement: S11 Fig — (A) Venn diagram of cancer-specific transcripts and non-cancerous liver specific transcripts. (B) Percentage of TE-derived exons in each item of the Venn diagram in A. P-values for the enrichment of TE-derived exons in cancer-specific transcripts and non-cancerous liver-specific transcripts were calculated by Fisher’s exact test. Comparisons were done for TE-derived exons in cancer-specific transcripts and other transcripts and for TE-derived exons in liver-specific transcripts and other transcripts. N.S.: Not significant. (PDF) [file pgen.1010342.s011.pdf]

S12 Fig.

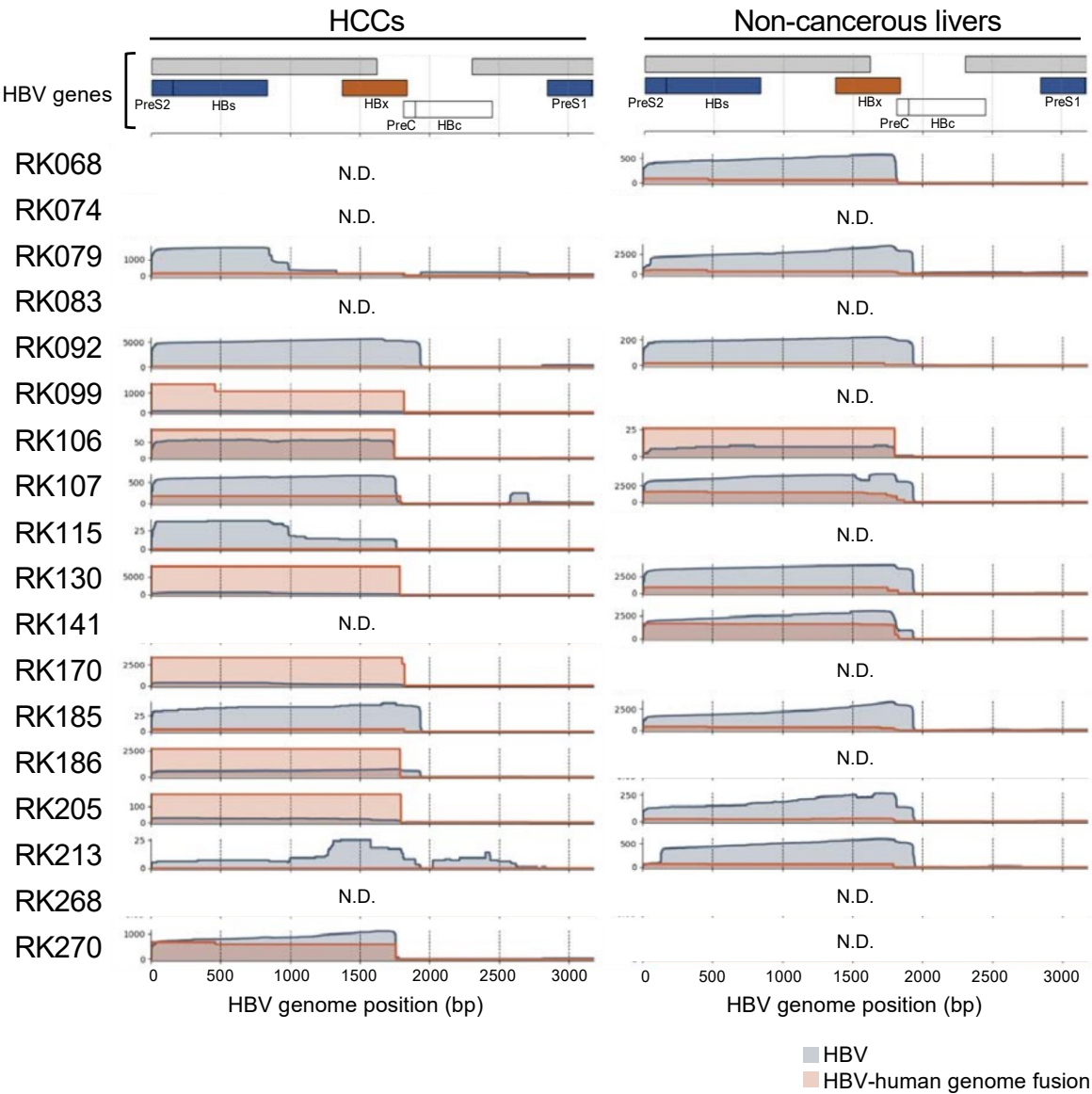

Supplement: S12 Fig — HBV transcripts were classified as “HBV (HBV alone)” and “HBV-human genome fusion”. (Upper panels) Schematic diagrams of the HBV genome structure. (Lower panels) RNA-seq coverage of HBV transcripts (HBV alone) and HBV-human genome fusion transcripts are shown in blue and red. N.D.: Not detected. (PDF) [file pgen.1010342.s012.pdf]

S13 Fig.

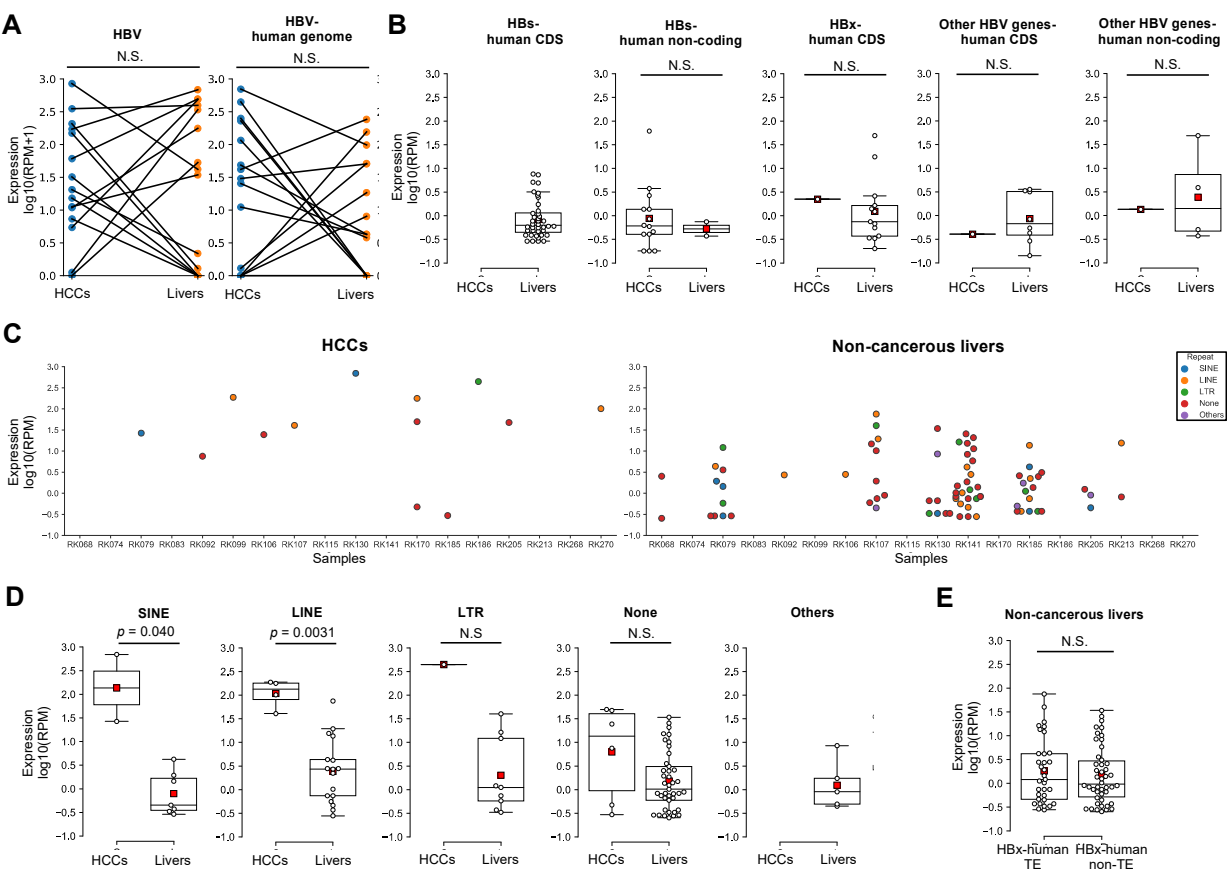

Supplement: S13 Fig — (A) Comparison of HBV transcript expression levels in cancer and matched non-cancerous livers. HBV transcripts were classified as “HBV (HBV alone)” and “HBV-human genome fusion”. Total expression levels of transcripts were plotted for each sample. Statistical significances were calculated by the Wilcoxon signed-rank test (B) Expression levels of HBV-human genome fusion transcripts by each transcript type in HCCs and non-cancerous livers. Statistical significances was calculated by the Wilcoxon rank-sum test. (C) Expression levels of HBx-human TE fusion transcripts. HBx-human non-coding fusion transcripts were classified according to the repeat types of the human genome. Their expression levels are shown for each sample. (D) Expression levels of HBx-human TE fusion transcripts in HCCs and livers. Statistical significances was calculated by the Wilcoxon rank-sum test. (E) Expression levels of HBx-human TE fusion transcripts and non-HBx human TE fusion transcripts in livers. Statistical significances was calculated by the Wilcoxon rank-sum test. (PDF) [file pgen.1010342.s013.pdf]

S14 Fig.

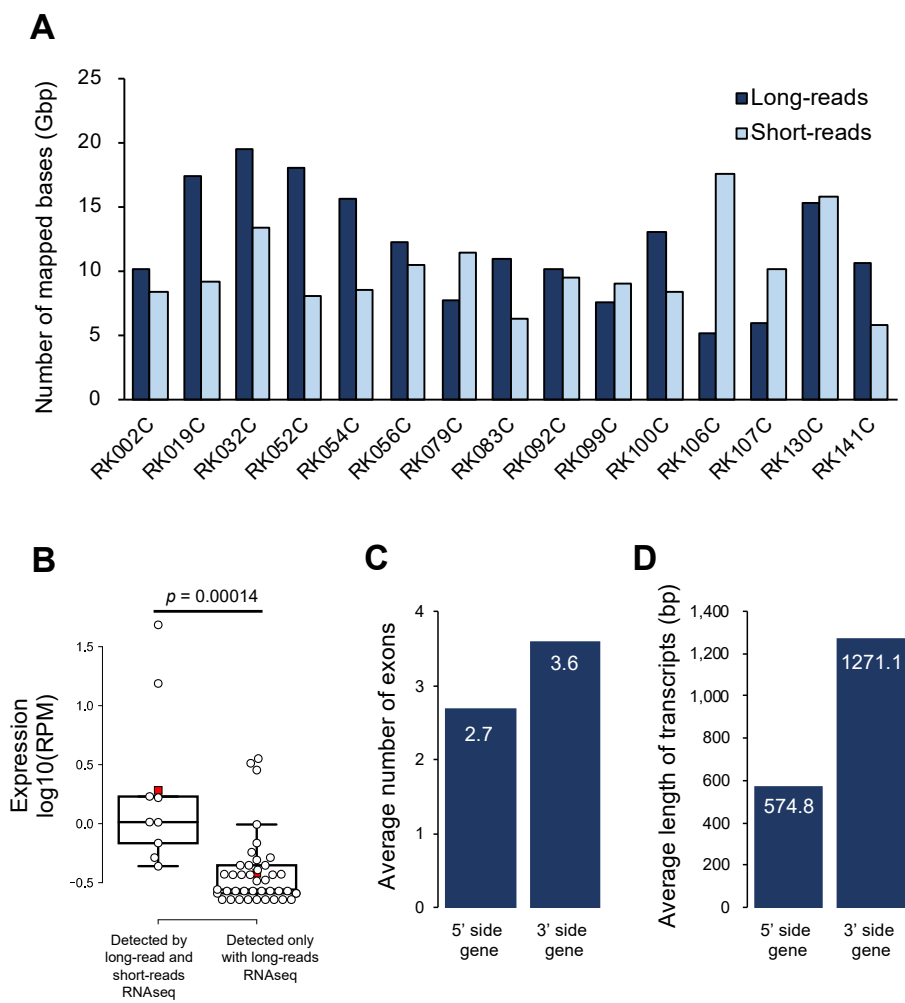

Supplement: S14 Fig — (A) Number of mapped bases between long-reads and short-reads RNA-seq. (B) Expression levels of fusion genes detected by both long-reads and short-reads RNA-seq and those detected by long-reads RNA-seq only. P-values were calculated by the Wilcoxon rank-sum test. (C) Average number of exons at the 5’ and 3’ sides of the fusion gene. (D) Average transcript length (bp) of the 5’ and 3’ sides of the fusion gene. (PDF) [file pgen.1010342.s014.pdf]

**S15 Fig.**

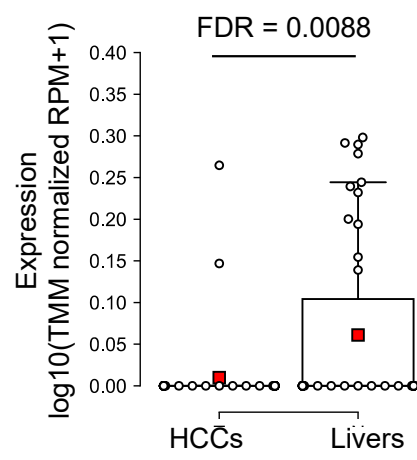

Supplement: S15 Fig — P-values were calculated by quasi-likelihood methods. The Benjamini-Hochberg method was used for multiple testing correction (FDR). (PDF) [file pgen.1010342.s015.pdf]

S16 Fig.

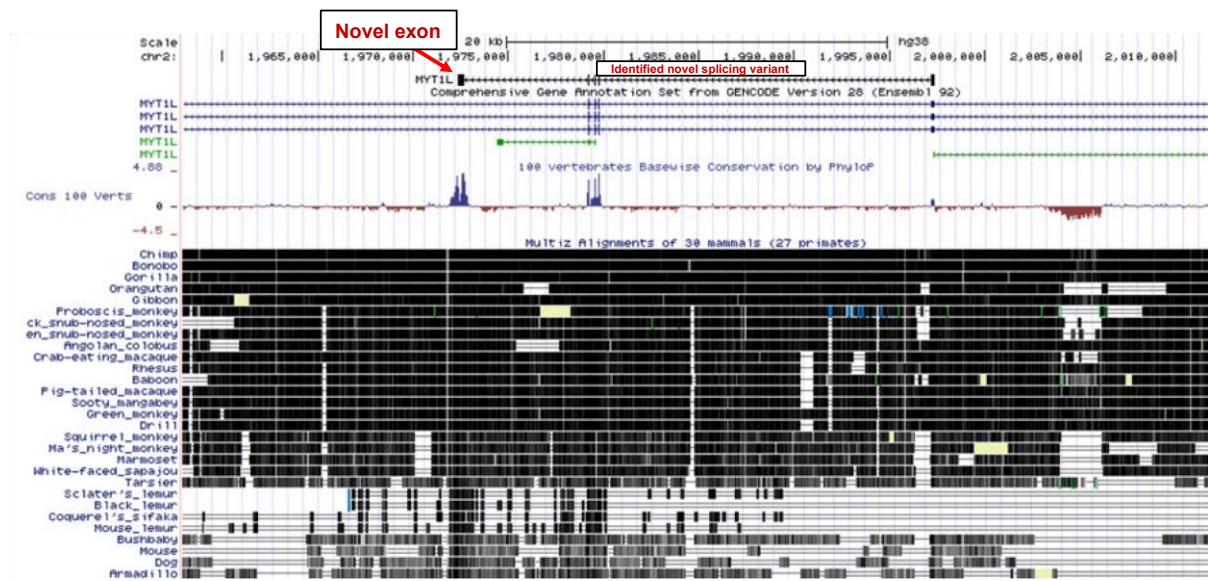

Supplement: S16 Fig — From top, a novel transcript, reference transcripts, and two types of multiple sequence comparisons are shown. (PDF) [file pgen.1010342.s016.pdf]

S17 Fig.

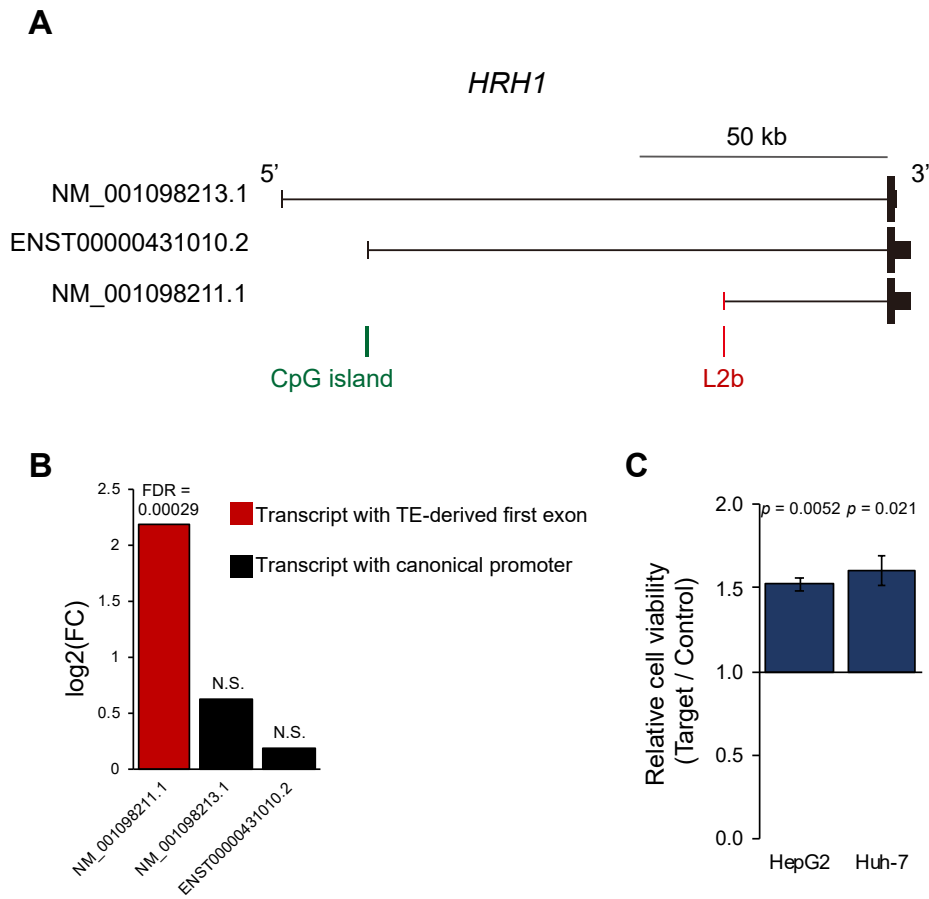

Supplement: S17 Fig — (A) Schematic diagram of the structural differences in L2-HRH1 (NM_001098211.1) and other HRH1 transcripts. (B) Bar chart showing the expression differences between HCCs and non-cancerous livers in HRH1 transcripts. P-values were calculated by quasi-likelihood methods. The Benjamini-Hochberg method was used for multiple testing correction (FDR). (C) Effect of L2-HRH1 overexpression on the proliferation of HepG2 and Huh-7 cell lines as assessed by CCK-8 expression. P-values were calculated by the one-sample t-test (n = 3). (PDF) [file pgen.1010342.s017.pdf]

S18 Fig.

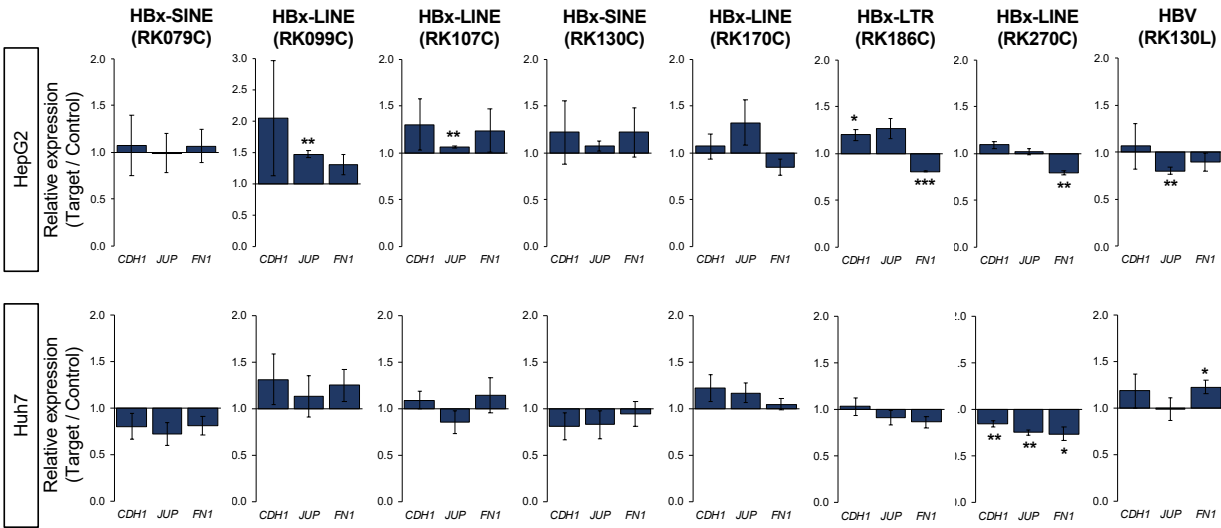

Supplement: S18 Fig — P-values were calculated by the one-sample t-test (*p < 0.1, **p < 0.05, ***p < 0.01). (PDF) [file pgen.1010342.s018.pdf]
