# Supplementary material for: Comprehensive analysis of full-length transcripts reveals novel splicing abnormalities and oncogenic transcripts in liver cancer
Source: PLoS Genet. 2022 Aug 4;18(8):e1010342. doi: 10.1371/journal.pgen.1010342 (PMC9380957; doi:10.1371/journal.pgen.1010342)
Supplement: S1 Table — (PDF) [file pgen.1010342.s019.pdf]

S1 Table

Items	Values
Total sequenced reads	16,538,905
Filtered reads	13,494,736
Filtered read bases (bp)	15,054,986,136
Longest read length	25,575
Mapped reads	13,424,802
Mapping rate (%)	99.48
Mapped bases	15,037,722,889
Average read length (bp)	1,120.15
Mismatch rate (%)	2
Insertion rate (%)	2
Deletion rate (%)	3
