# Supplementary material for: Comprehensive analysis of full-length transcripts reveals novel splicing abnormalities and oncogenic transcripts in liver cancer
Source: PLoS Genet. 2022 Aug 4;18(8):e1010342. doi: 10.1371/journal.pgen.1010342 (PMC9380957; doi:10.1371/journal.pgen.1010342)
Supplement: S2 Table — (PDF) [file pgen.1010342.s020.pdf]

S2 Table

Fusion gene	Sample	Forward primer	Reverse primer
BCAS3-BCAS4	MCF-7	CCTCCTGATGCTGCTCGT	CTCCTCCGTGTGCTCCAT
BCAS3-ATXN7	MCF-7	CAGTGGTGAAGCACAAGAGC	ATGTAGAGACGGTGGCTGCT
C8B-PAH	RK107C	GCCAATGCACCAACTTCTTC	CTCTGTGGCATCTCCTGTCA
CPS1-WNT10B	RK107C	GACAGAAAGGAGCCTGATGC	TCTGACAAGGGGACAGAACC
GCH1-SERPINA6	RK107C	TTGCTGGGAAACAACAAAGA	GGACAAGGGGAAGATGAACA
ABCD3-C1orf123	RK107C	AGACTCCTGGGCCTTTTCAT	CCAGACCAGCTGCTTACACA
AC138969.4-PDXDC1	RK107C	ACTTGTGCAGAGGGAGATGG	GCGGGAGGAGGAAGTAGAG
TBC1D23-TF	RK107C	GCTCTTTGTCTGGCCATCTC	AGAAGGGAGATGTGGCCTTT
NBEAL1-RPL12	RK107C	ATCTCGTTGGGGTCGAACTT	GGCAATGTCATCACCAACCT
