# Supplementary material for: Comprehensive analysis of full-length transcripts reveals novel splicing abnormalities and oncogenic transcripts in liver cancer
Source: PLoS Genet. 2022 Aug 4;18(8):e1010342. doi: 10.1371/journal.pgen.1010342 (PMC9380957; doi:10.1371/journal.pgen.1010342)
Supplement: S3 Table — (PDF) [file pgen.1010342.s021.pdf]

S3 Table

First exon (n = 500 for novel exons, n = 33,598 for known exons)

Repeat	Novel	Known	Odds ratio	*p*-value	Bonferroni adjusted *p*-value
SINE	41	138	21.66	9.60E-37	**4.80E-36**
LINE	72	205	27.40	1.66E-68	**8.29E-68**
LTR	49	184	19.73	8.17E-42	**4.09E-41**
SVA	0	3	0.00	1	1
DNA	5	41	8.27	0.000555002	**0.0028**

Last exon (n = 372 for novel exons, n = 30,838 for known exons)

Repeat	Novel	Known	Odds ratio	*p*-value	Bonferroni adjusted *p*-value
SINE	62	152	40.38	3.02E-68	**1.51E-67**
LINE	30	148	18.19	1.12E-25	**5.59E-25**
LTR	22	132	14.62	1.50E-17	**7.52E-17**
SVA	0	4	0.00	1	1
DNA	2	63	2.64	0.181584153	0.91

Middle exon (n = 129 for novel exons, n = 157,322 for known exons)

Repeat	Novel	Known	Odds ratio	*p*-value	Bonferroni adjusted *p*-value
SINE	26	1130	34.89	1.49E-29	**7.47E-29**
LINE	13	778	22.55	1.63E-13	**8.13E-13**
LTR	22	432	74.67	2.13E-32	**1.07E-31**
SVA	0	3	0.00	1	1
DNA	4	210	23.94	3.20E-05	**0.00016**
