# Supplementary material for: Comprehensive analysis of full-length transcripts reveals novel splicing abnormalities and oncogenic transcripts in liver cancer
Source: PLoS Genet. 2022 Aug 4;18(8):e1010342. doi: 10.1371/journal.pgen.1010342 (PMC9380957; doi:10.1371/journal.pgen.1010342)
Supplement: S4 Table — (PDF) [file pgen.1010342.s022.pdf]

S4 Table

DET-specific genes			DET and DEG genes			P-value	Odds ratio
BiExp genes	Non-Biexp gene	Percentage of BiExp genes	BiExp genes	Non-Biexp gene	Percentage of BiExp genes		
42	704	5.630	38	4274	0.881	1.591×10^{-15}	6.706
