# Supplementary material for: Comprehensive analysis of full-length transcripts reveals novel splicing abnormalities and oncogenic transcripts in liver cancer
Source: PLoS Genet. 2022 Aug 4;18(8):e1010342. doi: 10.1371/journal.pgen.1010342 (PMC9380957; doi:10.1371/journal.pgen.1010342)
Supplement: S5 Table — (PDF) [file pgen.1010342.s023.pdf]

S5 Table

※The total number of TE-derived exons in cancer-specific transcripts was 829

First exon

Position of the exon	Repeat	Repeat family	First exons of cancer-specific transcripts		First exons of transcripts that are not cancer-specific transcrips		Enrichment analysis of TE-derived exons in the first exon of cancer-specific transcripts and in the first exon of other transcripts					
							Sense			Antisense		
			Sense	Antisense	Sense	Antisense	Odds ratio	*p* -value	Bonferroni adjusted *p* -	Odds ratio	*p* -value	Bonferroni adjusted *p* -
first	DNA	TcMar-Mariner	0	1	0	0	nan	1.000	1.000	inf	0.133	1.000
first	DNA	TcMar-Tigger	3	3	11	4	1.782	0.418	1.000	4.901	0.054	1.000
first	DNA	hAT-Charlie	2	0	7	8	1.867	0.340	1.000	0.000	0.608	1.000
first	LINE	CR1	5	0	7	1	4.669	0.015	0.406	0.000	1.000	1.000
first	LINE	L1	17	49	36	78	3.092	3.333E-04	**0.00933**	4.136	8.120E-13	**2.274E-11**
first	LINE	L2	4	23	12	47	2.178	0.256	1.000	3.207	2.078E-05	**5.819E-04**
first	LTR	ERV1	12	11	34	26	2.309	0.016	0.453	2.768	0.007	0.194
first	LTR	ERVL	27	8	27	10	6.564	9.792E-11	**2.742E-09**	5.233	0.001	**0.034**
first	LTR	ERVL-MaLR	22	7	23	10	6.273	9.074E-09	**2.541E-07**	4.578	0.004	0.116
first	LTR	Gypsy	1	0	2	3	3.266	0.348	1.000	0.000	1.000	1.000
first	LTR	LTR	5	0	0	0	inf	4.119E-05	**0.00115**	nan	1.000	1.000
first	Retroposon	SVA	1	0	1	1	6.533	0.248	1.000	0.000	1.000	1.000
first	SINE	Alu	8	21	22	84	2.378	0.052	1.000	1.636	0.059	1.000
first	SINE	MIR	6	7	6	26	6.539	0.002	0.069	1.760	0.194	1.000

Last exon

Position of the exon	Repeat	Repeat family	Last exons of cancer-specific transcripts		Last exons of transcripts that are not cancer-specific transcripts		Enrichment analysis of TE-derived exons in the last exon of cancer-specific transcripts and in the last exon of other transcripts					
							Sense			Antisense		
			Sense	Antisense	Sense	Antisense	Odds ratio	*p* -value	Bonferroni adjusted *p* -	Odds ratio	*p* -value	Bonferroni adjusted *p* -
last	DNA	TcMar-Tigger	3	2	10	5	1.918	0.404	1.000	2.557	0.243	1.000
last	DNA	hAT-Blackjack	1	0	0	0	inf	0.135	1.000	nan	1.000	1.000
last	DNA	hAT-Charlie	2	1	22	6	0.581	0.763	1.000	1.065	1.000	1.000
last	DNA	hAT-Tip100	0	1	0	1	nan	1.000	1.000	6.391	0.252	1.000
last	LINE	L1	19	18	61	51	1.995	0.013	0.360	2.260	0.004	0.126
last	LINE	L2	1	1	15	10	0.426	0.713	1.000	0.639	1.000	1.000
last	LTR	ERV1	12	2	40	10	1.919	0.064	1.000	1.278	0.672	1.000
last	LTR	ERVK	5	1	14	2	2.284	0.167	1.000	3.195	0.354	1.000
last	LTR	ERVL	3	1	10	3	1.918	0.404	1.000	2.130	0.441	1.000
last	LTR	ERVL-MaLR	8	3	32	8	1.599	0.244	1.000	2.397	0.178	1.000
last	Retroposon	SVA	1	1	2	0	3.195	0.354	1.000	inf	0.135	1.000
last	SINE	5S-Deu-L2	2	0	1	0	12.785	0.050	1.000	nan	1.000	1.000
last	SINE	Alu	26	14	93	70	1.791	0.015	0.410	1.279	0.423	1.000
last	SINE	MIR	0	1	6	4	0.000	1.000	1.000	1.598	0.517	1.000

Middle exon

Position of the exon	Repeat	Repeat family	Middle exons of cancer-specific transcripts		Middle exons of transcripts that are not cancer-specific transcripts		Enrichment analysis of TE-derived exons in the middle exon of cancer-specific transcripts and in the middle exon of other transcripts					
							Sense			Antisense		
			Sense	Antisense	Sense	Antisense	Odds ratio	*p* -value	Bonferroni adjusted *p* -	Odds ratio	*p* -value	Bonferroni adjusted *p* -
middle	DNA	MULE-MuDR	1	0	1	0	4.747	0.318	1.000	nan	1.000	1.000
middle	DNA	TcMar-Mariner	0	1	3	4	0.000	1.000	1.000	1.187	1.000	1.000
middle	DNA	TcMar-Tigger	4	6	40	29	0.475	0.167	1.000	0.982	1.000	1.000
middle	DNA	hAT-Ac	1	0	7	6	0.678	1.000	1.000	0.000	0.599	1.000
middle	DNA	hAT-Charlie	10	6	46	40	1.032	0.861	1.000	0.712	0.560	1.000
middle	LINE	CR1	7	9	25	30	1.329	0.485	1.000	1.424	0.395	1.000
middle	LINE	L1	21	54	120	253	0.831	0.505	1.000	1.013	0.940	1.000
middle	LINE	L2	22	26	94	139	1.111	0.625	1.000	0.888	0.681	1.000
middle	LINE	RTE-BovB	4	0	12	6	1.582	0.504	1.000	0.000	0.599	1.000
middle	LINE	RTE-X	0	2	3	2	0.000	1.000	1.000	4.748	0.142	1.000
middle	LTR	ERV1	17	12	50	55	1.614	0.105	1.000	1.036	0.872	1.000
middle	LTR	ERVK	2	2	13	4	0.730	1.000	1.000	2.374	0.281	1.000
middle	LTR	ERVL	11	9	40	39	1.306	0.458	1.000	1.096	0.849	1.000
middle	LTR	ERVL-MaLR	26	26	77	90	1.603	0.050	1.000	1.372	0.176	1.000
middle	LTR	Gypsy	0	1	1	2	0.000	1.000	1.000	2.374	0.436	1.000
middle	SINE	5S-Deu-L2	1	0	2	2	2.374	0.436	1.000	0.000	1.000	1.000
middle	SINE	Alu	20	98	98	650	0.969	1.000	1.000	0.715	0.002	0.066
middle	SINE	MIR	16	37	75	176	1.013	1.000	1.000	0.998	1.000	1.000
middle	SINE	tRNA	1	3	2	14	2.374	0.436	1.000	1.017	1.000	1.000
middle	SINE	tRNA-Deu	0	1	0	0	nan	1.000	1.000	inf	0.174	1.000
