# Supplementary material for: Comprehensive analysis of full-length transcripts reveals novel splicing abnormalities and oncogenic transcripts in liver cancer
Source: PLoS Genet. 2022 Aug 4;18(8):e1010342. doi: 10.1371/journal.pgen.1010342 (PMC9380957; doi:10.1371/journal.pgen.1010342)
Supplement: S6 Table — (PDF) [file pgen.1010342.s024.pdf]

S6 Table

※The total number of TE-derived exons in non-cancerous liver-specific transcripts was 235

First exon

Position of the exon	Repeat	Repeat family	First exons of matced liver-specific transcripts		First exons of splicing variants that are not matched liver-specific transcripts		Enrichment analysis of TE-derived exons in the first exon of matched liver-specific transcripts and in the first exon of other transcripts					
							Sense			Antisense		
			Sense	Antisense	Sense	Antisense	Odds ratio	*p* -value	Bonferroni adjusted *p* -	Odds ratio	*p* -value	Bonferroni adjusted *p* -
first	DNA	hAT-Tip100	0	1	1	1	0.000	1.000	1.000	23.474	0.080	1.000
first	LINE	L1	6	6	46	120	3.069	0.019	0.303	1.174	0.649	1.000
first	LINE	L2	1	13	15	58	1.564	0.487	1.000	5.298	5.379E-06	**8.607E-05**
first	LTR	ERV1	2	5	44	31	1.066	0.713	1.000	3.794	0.015	0.239
first	LTR	ERVL	4	1	51	16	1.842	0.287	1.000	1.466	0.508	1.000
first	LTR	ERVL-MaLR	0	2	44	15	0.000	0.263	1.000	3.131	0.152	1.000
first	SINE	Alu	3	10	28	96	2.517	0.132	1.000	2.454	0.012	0.186
first	SINE	MIR	2	2	10	30	4.697	0.084	1.000	1.565	0.378	1.000

Last exon

Position of the exon	Repeat	Repeat family	Last exons of matced liver-specific transcripts		Last exons of splicing variants that are not matched liver-specific transcripts		Enrichment analysis of TE-derived exons in the last exon of matched liver-specific transcripts and in the last exon of other transcripts					
							Sense			Antisense		
			Sense	Antisense	Sense	Antisense	Odds ratio	*p* -value	Bonferroni adjusted *p* -	Odds ratio	*p* -value	Bonferroni adjusted *p* -
last	DNA	TcMar-Tigger	1	0	12	7	1.844	0.437	1.000	0.000	1.000	1.000
last	DNA	hAT-Charlie	1	1	23	6	0.962	1.000	1.000	3.688	0.266	1.000
last	LINE	CR1	0	1	2	0	0.000	1.000	1.000	inf	0.043	0.865
last	LINE	L1	4	5	75	63	1.180	0.586	1.000	1.758	0.220	1.000
last	LINE	L2	0	2	16	9	0.000	1.000	1.000	4.920	0.079	1.000
last	LTR	ERV1	1	2	50	10	0.442	0.727	1.000	4.428	0.093	1.000
last	LTR	ERVK	1	0	18	3	1.229	0.569	1.000	0.000	1.000	1.000
last	LTR	ERVL	0	1	13	3	0.000	1.000	1.000	7.377	0.162	1.000
last	LTR	ERVL-MaLR	4	2	36	9	2.462	0.093	1.000	4.920	0.079	1.000
last	SINE	Alu	13	5	106	78	2.729	0.002	**0.039**	1.419	0.411	1.000

Middle exon

Position of the exon	Repeat	Repeat family	Middle exons of matced liver-specific transcripts		Middle exons of transcripts that are not matched liver-specific transcripts		Enrichment analysis of TE-derived exons in the middle exon of matched liver-specific transcripts and in the middle exon of other transcripts					
							Sense			Antisense		
			Sense	Antisense	Sense	Antisense	Odds ratio	*p* -value	Bonferroni adjusted *p* -	Odds ratio	*p* -value	Bonferroni adjusted *p* -
middle	DNA	TcMar-Tigger	1	1	43	34	0.359	0.522	1.000	0.455	0.722	1.000
middle	DNA	hAT-Charlie	2	3	52	42	0.595	0.772	1.000	1.104	0.753	1.000
middle	LINE	CR1	0	4	29	33	0.000	0.419	1.000	1.874	0.284	1.000
middle	LINE	L1	6	16	133	283	0.697	0.479	1.000	0.874	0.716	1.000
middle	LINE	L2	3	12	110	149	0.421	0.165	1.000	1.245	0.411	1.000
middle	LTR	ERV1	4	6	57	59	1.085	0.787	1.000	1.573	0.290	1.000
middle	LTR	ERVL	0	2	50	46	0.000	0.074	1.000	0.672	1.000	1.000
middle	LTR	ERVL-MaLR	10	7	91	103	1.700	0.138	1.000	1.051	0.841	1.000
middle	SINE	Alu	7	34	109	704	0.993	1.000	1.000	0.746	0.104	1.000
middle	SINE	MIR	6	8	80	201	1.160	0.651	1.000	0.615	0.194	1.000
middle	SINE	tRNA	0	1	3	17	0.000	1.000	1.000	0.909	1.000	1.000
