# Supplementary material for: Comprehensive analysis of full-length transcripts reveals novel splicing abnormalities and oncogenic transcripts in liver cancer
Source: PLoS Genet. 2022 Aug 4;18(8):e1010342. doi: 10.1371/journal.pgen.1010342 (PMC9380957; doi:10.1371/journal.pgen.1010342)
Supplement: S7 Table — (PDF) [file pgen.1010342.s025.pdf]

S7 Table

Fusion genes	Sample	Forward primer	Reverse primer
SLC20A2-SENP6	RK059C	GCTCCGGGGCTCTTTACC	TATCTACGTGCCGGGTCCAT
UBE3D-SENP6	RK119C	ACCTCTCCTGGTCCTGTTGA	TGGTCCAGTAGACTCATTCAAAGT
TBRG1-IFT46	RK130C	GATCATCACCGACCGACCTG	GCATCAGCGTGTCAATGTCG
ATL3-TBRG1	RK116C	CGTTTTCGGGTGAAACTTGACA	AGGCAGTGTTTTTCAAGGTGT
SDC2-CPQ	RK107C	AAACTTCTGCCGTAGCTCCC	GCTTTAGCAACATCTCCACAGC
SDC2-CSAD	RK116C	TCCAGGCTAAGGACTTTTGTT	AACATTGCGCAGGTCACTGT
ABCD3-C1orff123	RK107C	GCTAATCCAGACCAGCTGCT	AAAGGAACGAGCCTCTGTGG
ABCD3-DST	RK076C	GCTGTGGTGGACAAGGTGTT	TCTGGCAGCTGCAGTTGTTA
LGSN-SLC13A3	RK059C	ACAGTCACCCACCTAGGAGG	TGATGAGCAGCACAGCCAG
LGSN-GGPS1	RK052C	ACAGTCACCCACCTAGGAGG	GTCCTCTGGAACTTTCAGCCA
LDLR-SLC41A2	RK124C	GGCTGAGCTGGATCACTTGA	CGGGCTTCTTTAAAAGAGCAA
CLN3-LDLR	RK170C	GGAGAGCAGTGGCACAATCT	ACAGTGTTTTGTCCCTGGGG
