# Supplementary material for: Comprehensive analysis of full-length transcripts reveals novel splicing abnormalities and oncogenic transcripts in liver cancer
Source: PLoS Genet. 2022 Aug 4;18(8):e1010342. doi: 10.1371/journal.pgen.1010342 (PMC9380957; doi:10.1371/journal.pgen.1010342)
Supplement: S8 Table — (PDF) [file pgen.1010342.s026.pdf]

S8 Table

Pathway name	Entities found	Entities total	Entities ratio	Entities *p*-value	Entities FDR
Interferon alpha/beta signaling	65	191	0.0130	0.0000	1.10E-14
Antigen Presentation: Folding, assembly and peptide loading of class I MHC	38	102	0.0069	0.0000	1.10E-14
ER-Phagosome pathway	42	165	0.0112	0.0000	1.10E-14
Endosomal/Vacuolar pathway	36	82	0.0056	1.11E-16	1.10E-14
Interferon Signaling	66	401	0.0272	1.11E-16	1.10E-14
Antigen processing-Cross presentation	43	187	0.0127	1.11E-16	1.10E-14
Interferon gamma signaling	45	255	0.0173	1.11E-16	1.10E-14
Immunoregulatory interactions between a Lymphoid and a non-Lymphoid cell	39	317	0.0215	1.11E-16	1.10E-14
Cytokine Signaling in Immune system	82	1108	0.0753	1.11E-16	1.10E-14
Class I MHC mediated antigen processing & presentation	44	465	0.0316	5.00E-15	4.45E-13
Immune System	122	2713	0.1843	2.94E-14	2.38E-12
Adaptive Immune System	56	1003	0.0681	1.08E-09	8.00E-08
