# Supplementary material for: Comprehensive analysis of full-length transcripts reveals novel splicing abnormalities and oncogenic transcripts in liver cancer
Source: PLoS Genet. 2022 Aug 4;18(8):e1010342. doi: 10.1371/journal.pgen.1010342 (PMC9380957; doi:10.1371/journal.pgen.1010342)
Supplement: S9 Table — (PDF) [file pgen.1010342.s027.pdf]

S9 Table

Pathway name	Entities found	Entities total	Entities ratio	Entities *p*-value	Entities FDR
Interferon alpha/beta signaling	6	191	0.0130	1.6E-04	0.0330
Iron uptake and transport	4	83	0.0056	4.3E-04	0.0330
The citric acid (TCA) cycle and respiratory electron transport	6	233	0.0158	4.5E-04	0.0330
RUNX3 Regulates Immune Response and Cell Migration	2	10	6.79E-04	8.5E-04	0.0451
Nef mediated downregulation of MHC class I complex cell surface expression	2	11	7.47E-04	0.00102	0.0451
