# Supplementary material for: Comprehensive analysis of full-length transcripts reveals novel splicing abnormalities and oncogenic transcripts in liver cancer
Source: PLoS Genet. 2022 Aug 4;18(8):e1010342. doi: 10.1371/journal.pgen.1010342 (PMC9380957; doi:10.1371/journal.pgen.1010342)
Supplement: S10 Table — (PDF) [file pgen.1010342.s028.pdf]

S10 Table

Summary of RNA-seq and mapping of MCF-7

Items	v3.0.3	v6.0.6
Total sequenced reads	1,000,000	1,000,000
Filtered reads	926,530	989,031
Filtered read bases (bp)	1,035,718,911	1,105,567,703
Longest read length	16,997	17,151
Mapped reads	921,744	983,477
Average_length	1,122.33	1,122.49
Mismatch_rate (%)	2.00	1.45
Insertion_rate (%)	2.03	1.24
Deletion_rate (%)	2.78	1.87

Result of splicing variant analysis

Items	v3.0.3	v6.0.6
Number of known transcripts	8,919	9,228
Number of novel transcripts	410	446

Result of fusion analysis

Items	v3.0.3	v6.0.6
Fusion genes (Number of support reads)	*BCAS3-BCAS4* (3)	*BCAS3-BCAS4* (4),<br>*BCAS3-ATXN7* (4)

※ In v3.0.3, the number of support reads for *BCAS3-ATXN7* was 2.
