# Supplementary material for: Comprehensive analysis of full-length transcripts reveals novel splicing abnormalities and oncogenic transcripts in liver cancer
Source: PLoS Genet. 2022 Aug 4;18(8):e1010342. doi: 10.1371/journal.pgen.1010342 (PMC9380957; doi:10.1371/journal.pgen.1010342)
Supplement: S11 Table — (PDF) [file pgen.1010342.s029.pdf]

S11 Table

Target transcript	Clone type	Sample	Forward Sequence	Reverse sequence	Target size (bp)
L1-MET (XM_006715990.2)	CDS	RK030C	ATGGGTCAATTCAGCGAAGT	CTATGATGTCTCCCAGAAGG	2883
MET (NM_001127500.2)	CDS	pFN21AB5617	ATGAAGGCCCCCGCTGTGCTT	CTATGATGTCTCCCAGAAGG	4227
L2-HRH1 (NM_001098211.1)	CDS	RK048C	ATGAGCCTCCCCAATTCCTC	TTAGGAGCGAATATGCAGAA	1464
HBx-SINE_079C	Full length	RK079C	TCCACAACATTTCACCGAGC	TTTGTTTGTTTTCCTTTTGCAG	2786
HBx-LINE_099C	Full length	RK099C	TCCACAACCTTCCACCAAAC	GTCTCACTTTAATACAGGTTCA	2394
HBx-LINE_107C	Full length	RK107C	CAGGCCATGCAGTGGAACTC	TGGAACACAGCCACACCCAT	2552
HBx-SINE_130C	Full length	RK130C	TCCACCACATTCCACCAAGC	TTGTGTTCAGTCTTGAGTCT	2011
HBx-LINE_170C	Full length	RK170C	TCCACAACATTCCACCAAAC	TTAGAAAACTGAATTAATTTA	1973
HBx-LTR_186C	Full length	RK186C	TCCACAACATTCCACCAAGC	TAGGAGGGATGGACTTTAAT	2600
HBx-LINE_270C	Full length	RK270C	TCCACAACATTCCACCAAGC	TTTGAATCTTTTCAGATCTTCAC	1946
HBV_130L	Full length	RK130L	TCCACCACATTCCACCAAGC	GGTCAATGTCCATGCCCCAA	1998
