# Supplementary material for: Comprehensive analysis of full-length transcripts reveals novel splicing abnormalities and oncogenic transcripts in liver cancer
Source: PLoS Genet. 2022 Aug 4;18(8):e1010342. doi: 10.1371/journal.pgen.1010342 (PMC9380957; doi:10.1371/journal.pgen.1010342)
Supplement: S12 Table — (PDF) [file pgen.1010342.s030.pdf]

S12 Table

siRNA design

siRNA ID	Target Name	Sense/Antisense	siRNA design	Start on Target	Target sequence
siL1-*MET* #1	*MET* _XM_006715990.2	s	GCGGGACUCCGUGGGCGUAdTdT	31	GCGGGACTCCGTGGGCGTA
siL1-*MET* #1	*MET* _XM_006715990.2	a	UACGCCCACGGAGUCCCGCdTdT	31	TACGCCCACGGAGTCCCGC
siL1-*MET* #2	*MET* _XM_006715990.2	s	GAGCCUAGGCUUAGUCCUAdTdT	109	GAGCCTAGGCTTAGTCCTA
siL1-*MET* #2	*MET* _XM_006715990.2	a	UAGGACUAAGCCUAGGCUCdTdT	109	TAGGACTAAGCCTAGGCTC

qPCR primers

Target	Forward/Reverse	length (bp)	Sequence
L1-*MET*	F	20	GAGCCAGGCAGAAAATGTGC
L1-*MET*	R	20	CTTCAGTGCAGGGCTAGGAC
