# Supplementary material for: Comprehensive analysis of full-length transcripts reveals novel splicing abnormalities and oncogenic transcripts in liver cancer
Source: PLoS Genet. 2022 Aug 4;18(8):e1010342. doi: 10.1371/journal.pgen.1010342 (PMC9380957; doi:10.1371/journal.pgen.1010342)
Supplement: Attachment — Submitted filename: full-revision_220522.fuji.docx [file pgen.1010342.s043.docx]

**Manuscript number:** RC-2022-01240

**Corresponding author(s):** Akihiro, Fujimoto

*[Please use this template only if the submitted manuscript should be considered by the affiliate journal as a full revision in response to the points raised by the reviewers.*

*If you wish to submit a preliminary revision with a revision plan, please use our "*[*Revision Plan*](https://docs.google.com/document/u/0/d/1HHp62Tfeb44nd3Gu_3ChB0b8gmMdEog7GjZcZFwHSiQ/edit)*" template.* ***It is important to use the appropriate template to clearly inform the editors of your intentions.****]*

## 1. General Statements [optional]

Dear Editor,

We greatly appreciate the reviewers’ very insightful comments. To address the comments, we added results of benchmarking. We also modified figures and descriptions, and released the Docker file for our tool. Thanks to the comments, we believe that we have added important information and made our paper more readable.

Once again, we are profoundly appreciative of the reviewers’ comments and advice.

Sincerely,

Akihiro Fujimoto, Ph.D.

Professor

Department of Human Genetics

Graduate School of Medicine

The University of Tokyo

7-3-1 Hongo, Bunkyo-ku, Tokyo, 113-0033, Japan

E-mail: [afujimoto@m.u-tokyo.ac.jp](mailto:afujimoto@m.u-tokyo.ac.jp)

## 2. Point-by-point description of the revisions

*This section is mandatory.* *Please insert a point-by-point reply describing the revisions that were already carried out and included in the transferred manuscript.*

*Reviewer #1: General comments:*

*Fujimoto and collaborators use Nanopore-based cDNA sequencing for genome-wide transcriptome analysis of a collection of hepatocellular carcinomas (HCCs) and matched normal liver tissues. To improve detection of alternatively spliced isoforms and hybrid transcripts potentially deriving from genomic rearrangements, they develop a dedicated pipeline SPLICE, which they benchmark against available software used for the same analysis. Besides having dual functionality (calls both alternative transcripts and fused transcripts), SPLICE seems to outperform previous software in calling alternative/fused transcripts and accuracy. They use the SPLICE pipeline to call isoforms and gene fusions in normal liver cells and HCCs and perform basic functional validations on novel fusions identified.
The manuscript is well written, and the analyses are well performed. Perhaps the benchmarking of the SPLICE pipeline could have been more extensive (i.e., performed on additional independent datasets).*

*Major points:
1. Line 149-150: "We compared the results of mapping to the reference genome and the reference transcriptome sequences, and removed candidates if both were inconsistent (removal of mapping errors). " Please specify what "both were inconsistent" means.*

Our reply; Thank you for this comment. The accuracy of fusion gene detection is influenced by mapping errors. To remove possible mapping errors, SPLICE aligned reads to the reference genome and the reference transcriptome sequences and compared the results. If the results are inconsistent (for example, *GeneA-GeneB* in the reference genome and *GeneA-GeneB* in the transcriptome genome, or *GeneA-GeneB* in the reference genome and *GeneA* in the transcriptome genome), SPLICE considers the candidates as false positive and removes them from the analysis.

We changed the sentence “We compared the results of mapping to the reference genome and the reference transcriptome sequences, and removed candidates if both were inconsistent (removal of mapping errors).” to “we compared the results of mapping to the reference genome and the reference transcriptome sequences and removed candidates if both results did not detect the same fusion genes (removal of mapping errors).” (line 163-165).

*2. Concerning TE-derived novel exons, in principle, this may lead to altered expression of the TE-transcript (as the Authors report for L1-MET) or to altered splicing of the transcript (i.e., other exon/introns could be retained or excluded). Can the Authors assess whether the inclusion of the TE in a transcript enhances its expression or affects the splicing of the "parental" transcript? If so, can they verify if the position of the insertion of the TE has any effect on expression and splicing?*

Our reply; Thank you very much for this important comment. As the reviewer mentioned, exonization of TE may affect the splicing patterns and gene expression levels of transcripts. To determine the effect of TE on expression levels, we compared the expression levels of transcripts with TE-derived novel exons with those of known transcripts of the gene. We found that the expression levels of transcripts with TE-derived novel exon were lower than those of known transcripts (Figure 1 in the reply). Since the same results were observed in all novel transcripts (Fig. 1E,F), most TE exonization would not affect the expression level of transcripts.

We then analyzed the effects of TE in the splicing change, we compared the numbers of novel splicing junctions between transcripts with TE-derived novel exons and other transcripts in each gene. The proportions of genes with novel splicing junctions were not significantly different between the transcripts with TE-derived novel exons and others (transcripts with TE-derived novel exons; 9.1% and others; 11.9%) (Figure 2 in the reply). As observed in L1-*MET* and L2-*RHR1*, transposons can affect expression levels and structures of transcripts, however, their effect would be limited to a part of genes.

Figure 1

Comparison of expression levels of transcripts with TE-derived novel exon and known transcripts. Only transcripts derived from genes with TE-derived novel exons were compared. The total number of transcripts is shown below the plot. Transcript abundance was measured in reads per million reads (RPM), and log10 converted values for RPM were shown in the violinplot. *P-values* were calculated by Wilcoxon rank-sum test.

Figure 2

Comparison of the percentage of novel splicing junction in transcripts with novel TE-derived exon and other transcripts. The total number of genes are shown below the plot. Transcripts with TE-derived novel exons and other transcripts were compared. *P-value* was calculated by Fisher’s exact test.

*3. Can the Authors explain why the NBEAL1-RPL12 was not detected by SPLICE?*

Our reply; Thank you for this comment. Although *NBEAL1-RPL12* fusion was detected by SPLICE, mapping results to the reference genome and the reference transcriptome were inconsistent and removed from the final result. As *NBEAL1-RPL12* was not validated by PCR (Supplemental Fig. S4B) (line 194-195), we consider that this fusion-gene is a false-positive, and filtering of SPLICE successfully removed false-positive fusions.

*4. Line 332: Can the Authors explain how the total amount of HVB mRNA was determined in each sample? Is it a relative amount calculated from the sequencing data? If so, it should be made clear in the text that this is a fractional measure.*

Our reply; Thank you very much for this comment. Expression levels were calculated by log10 converted reads per million reads (log10(RPM)) for each sample. We added the following sentences to the "Expression from HBV" subsection in the Results (line 345-346); “Expression levels were estimated by log10 converted support reads per million reads (log10(RPM)) for each sample.”.

*5. Fig4a: please specify if the y-axis "number of support reads" reports library normalized values.*

Our reply; Thank you for this comment. The values of the y-axis are row read counts. We added the following sentences to the Figure legend (line 355-356); “The y-axis shows the total number of support reads (raw counts)”.

*6. HCCs have more HBV-human genome fusion transcripts than normal liver. Could the authors clarify if these HCC transcripts are selectively found in tumors? or whether they are also expressed in normal liver samples? The paragraph starting from line 356 is confusing, and it is difficult to retrieve the above information for both HBs and HBx fusions.*

Our reply; We apologize for the confusing description. All HBV-human genome fusion transcripts were selectively expressed in the respective tissues. We added the following sentence to the "Expression from HBV" subsection in the Results (line 370-371); “Thirty and 146 HBV-human genome fusion transcripts were detected in the HCCs and matched livers (Supplemental Data S8), and all were selectively expressed in the respective tissues.”.

*7. Figure 4C: what was the control used to calculate the relative viability in these analyses?*

Our reply; Thank you for this comment. Fig. 4C shows the number of HBV-human fusion transcripts in the six categories. If this comment refers to Fig. 4H, cell lines transfected with an empty vector (pIRES2-AcGFP1-Nuc) was used as controls. This has been described in the "Gene overexpression" subsection of Methods (line 720-721).

*8. MYT1L: the Authors report the identification of a novel MYT1L transcript downregulated in HCC, and argue it may have a potential tumor-suppressive function. For the sake of clarity, it will be advisable to show also the differential expression (HCC vs. Liver) of the other transcripts expressed from the same locus.*

Our reply; Thank you for this important comment. In HCCs and normal livers, only a transcript with the novel exon was expressed. We changed the sentence “In the *MYT1L* gene, a highly-conserved novel exon was detected (Fig. 2E), and this transcript was significantly down-regulated in the HCCs” to “In the MYT1L gene, for example, a highly-conserved novel exon was detected (Fig. 2E), and only a transcript with the novel exon was expressed. This transcript was significantly down-regulated in HCCs” (line 475-476).

*Minor points:
1. Table S4: there is a typo, correct “secific” in “specific”*

Our reply; Thank you very much for this comment. We corrected the typo of Table S4.

*Reviewer #2: General comments:

Summary: This is both a presentation of a pipeline for analysis of Nanopore RNA-seq data, as well as an analysis of a cohort of 44 hepatocellular carcinomas against matched-normal liver tissue. It presents a number of quite intriguing results from the long-read RNA analysis, and suggests potential new targets for study in HCC. It is also worth noting that the current version of guppy (6) has functionality to detect primer sequences in the middle of reads and split those reads, which may obviate one of the steps in SPLICE.*

*Major comments:

1) The work done in this study used data that was basecalled using guppy 3.0.3. Since that version, I am aware of at least two major upgrades to the base caller accuracy, which would likely also improve the accuracy of isoform resolution. Given that the data is relatively low-coverage and that you have an automated workflow for the analysis, I would recommend re-basecalling using an updated basecaller and re-running your analysis using that. This is especially important given your comments in the paper about splice site misalignment.*

Our reply; Thank you very much for this important comment. We performed basecalling of a sequence data of MCF7 using the latest guppy v6.0.6 and compared the result with that by guppy v3.0.3. We randomly extracted 1M reads from MCF-7 reads that passed qscore filtering in guppy basecaller. The same reads were extracted and basecalled by guppy v3.0.3. These two data were analyzed by SPLICE.

The average error rate was 4.6 % for v6.0.6 and 6.8 % for v3.0.3. The number of transcripts was 9,674 for v6.0.6 and 9,329 for v3.0.3. Of these, the number of novel transcripts was 446 and 410, respectively. The number of fusion genes was 2 (*BCAS3-BCAS4*, and *BCAS3-ATXN7*) by v6.0.6 and one (*BCAS3-BCAS4*) by v3.0.3. As the reviewer mentioned, we found that using the latest version of guppy improved the accuracy and detected a larger number of transcripts.

We added the results to Supplemental Table S12. We also changed the sentences from “Second, our analysis removed the change of splicing sites within 5 bp to remove alignment errors (Fig. 1B). We consider that this cutoff value is necessary due to currently available high-error reads (Supplemental Data S2). However, sequencing technologies and basecallers are improving, and in the near future, we should be able to use a smaller cutoff value and identify larger numbers of splicing changes.” to “Second, the accuracy of the analysis depends on the sequencing error rate. Although several filters were applied to currently available high-error reads (Fig. 1B and Supplemental Fig. S1), sequencing errors can still affect the accuracy of the result. With improvements in sequencing technologies and basecallers, the identification of larger numbers of splicing changes with high accuracy should be possible in the future (Supplemental Table S10)” (line 542-546).

*2) You have compared your software to another tool for isoform analysis on Nanopore sequencing data, TALON. But a number of other tools exist for this purpose, including stringtie2, flair and bambu. My own testing has shown that stringtie2 outperforms TALON in terms of concordance with Illumina RNA-seq. It is quite important that you perform a complete comparison of your software to the state of the art for this purpose.*

Our reply; Thank you very much for this important comment. We compared our tool with four widely adopted long-reads RNA-seq analysis pipelines (TALON, FLAIR, StringTie, and bambu). For this comparison, we used sequence data of MCF-7 and HCC (RK107C). We randomly extracted 1 M reads from MCF-7 and an HCC (RK107C) sequence data using Seqtk (v1.3) (params: sample -s1 1000000). Reads were mapped to the reference genome sequence (hg38) with minimap2 (v2.17) (params: -ax splice --MD), and the output SAM files were converted to BAM files and sorted with samtools (v1.7) (Li et al. 2009).

For the benchmarking of TALON (v5.0), we corrected aligned reads with TranscriptClean (v2.0.3) (Wyman and Mortazavi 2018). Next, we ran the *talon_label_reads* module to flagging reads for internal priming (params: --ar 20). The TALON database was initialized by running the *talon_initialize_database* module (params: --l o --5p 500 --3p 300). Then, we ran the talon module to annotate the reads (params: --cov 0.8 --identity 0.8). To output transcript abundance, we first obtained a whitelist using the *talon_filter_transcripts* module (params: --maxFracA 0.5 --minCount 5), and then quantified transcripts using the *talon_abundance* module based on the whitelist. For the benchmarking of FLAIR (v1.5), the sorted BAM file was converted to BED12 using *bin/bam2Bed12.py*. We then corrected misaligned splice sites with the *flair-correct* module. High-confidence isoforms were defined from the corrected reads using the *flair-collapse* module (params: -s 3 --generate_map). For benchmarking of StringTie (v2.2.1), *Stringtie* was performed with input files consisting of long-read alignment and reference annotations (params: -L -c 3). For the benchmarking of Bambu (v2.0.0), *Bambu* was performed with input files consisting of long-reads alignments, reference annotations, and the reference genome (hg38) (params: min.readCount = 3). Candidates with low expression levels (support reads < 3), antisense, and intergenic transcripts were excluded from the comparison.

As a result, SPLICE identified the third highest number of transcripts after FLAIR and StringTie (Supplemental Fig. S3A). For MCF-7, SPLICE had the highest concordance rate with IsoSeq MCF-7 transcriptome data for known transcripts and second highest for novel transcripts (Supplemental Fig. S3B). These results indicate that SPLICE has sufficient accuracy for analyzing transcript aberrations.

We added the text to the "Comparison of SPLICE method with other tools" subsection of the Results (line 177-188) and the "Benchmarking" subsection of the Methods (line 644-669). We added the results to Supplemental Fig. S3.

*3) Likewise, for fusion detection, you compare to LongGF. You should also compare to (and cite) JAFFAL.*

Our reply; Thank you very much for this important comment. We compared the fusion genes detected from MCF7 by SPLICE, LongGF, and JAFFAL. We used 1 M reads randomly extracted from the MCF-7 and HCC (RK107C) sequence data as described above.

For the benchmarking of LongGF (v0.1.2), reads were mapped to the reference genome sequence (hg38) with minimap2 (v2.17) (params: -ax splice --MD), and the output SAM files were converted to BAM files and sorted with samtools (v1.7). We then ran the *longgf* module and obtained the list of fusion genes (params: min-overlap-len 100 bin_size 50 min-map-len 200 pseudogene 0 secondary_alignment 0 min_sup_read 3). For the benchmarking of JAFFAL (v2.2), we ran the *JAFFAL.groovy* module with zipped fastq files. In this comparison, close gene pairs (< 200,000 bp) were not considered, because they cannot be distinguished from read-through transcripts (Akiva et al. 2006).

As a result, previously known *BCAS3-BCAS4* (Edgren et al. 2011) was detected only by SPLICE, and *BCAS3-ATXN7* was detected by SPLICE and LongGF (Supplemental Fig. S4A,B). In HCC, *AC138969.4-PDXDC1* was detected only by SPLICE, *C8B-PAH* was detected by SPLICE and JAFFAL, three fusions (*GCH1-SERPINA6*, *ABCD3-C1orf123*, and *CPS1-WNT10B*) were detected by SPLICE and LongGF, and two fusions (*TBC1D23-TF* and *NBEAL1-RPL12*) were detected only by LongGF. One of the fusions (*NBEAL1-RPL12*), detected only by LongGF, cound not be validated by PCR (Supplemental Fig. S4B). These results indicate that SPLICE had the highest accuracy for detecting fusion genes.

We added the text to the "Comparison of SPLICE method with other tools" subsection in the Results (line 189-197) and the "Benchmarking" subsection in the Methods (line 671-683). We showed the results in Supplemental Fig. 4.

*4) In terms of the source code, I have questions. Why did you use BASH to run the Python code, instead of making this into a Python package? Why did you not use the functionality already available in BioPython for a number of basic sequence data handling tasks? Why is there not even a single function defined anywhere, let alone classes?*

*At some level, if it works, it works. But I have serious concerns about the long-term maintainability of the code in its current state.*

Our reply; Thank you very much for this critical comment. As the reviewer mentioned, we think it is better to make a python package and use BioPython for maintenance and long-term maintainability of the code. We provided a Docker package (see the reply to comment 5)), and this would promote usability.

*5) Also related to the code, it is generally the standard now to create a BioConda package or Docker container for a bioinformatics package. BioConda has the advantage that the BioContainers project automatically generate Docker and Singularity containers from it. Please provide one of these.*

Our reply; Thank you very much for this critical comment. We made a Docker file and provided it from our github page. It is available from the "Installation and usage via Docker" section.

*6) There is some quite nice functional validation work done on some of the DE transcripts that would have been hidden in a gene-level analysis. There is also some nice work on detecting HBV fusion genes. These both contain important results which are not mentioned at all in the abstract. I feel like the abstract as it stands is selling the paper short.*

Our reply; Thank you very much for this important comment. We added the following sentences to the abstract; “A comparison of expression levels identified 9,933 differentially expressed transcripts (DETs) in 4,744 genes. Interestingly, 746 genes with DETs, including the *LINE1-MET* transcript, were not found by a gene-level analysis. We also found that fusion transcripts of transposable elements and hepatitis B virus (HBV) were overexpressed in HCCs. *In vitro* experiments on DETs showed that LINE1-MET and HBV-human transposable elements promoted cell growth.”.

*7) Fig 5C shows a Venn diagram of fusions detected by short-read vs long-read sequencing, in which there is quite low overlap between these. You make the statement in the paper that "a combination of short- and long-reads can detect more fusion genes". I find it more likely that the short-read ICGC data had much greater depth of coverage than the MinION data you produced, which allowed for the detection of fusions that were expressed at much lower levels. This could be easily tested by downsampling the ICGC data to the same amount of sequence data as was generated on the MinION, and re-creating the Venn diagram with the fusions detected that way.*

Our reply; Thank you very much for this very important comment. We compared the amount of data between our long-reads and the previous short-reads. However, the amounts of data were not quite different (Supplemental Fig. S14A). Therefore, differences in depth are not likely to be the cause of the low overlap. We considered two explanations for the low overlap. Since most of the fusion genes missed by short-reads had very low expression levels (Supplemental Fig. S14B), many fusion genes with low expression levels were missed by the single approach. In addition, 28.9% of transcripts in long-reads lacked the 5' region (Supplemental Fig. S5 and Supplemental Fig. S14C, D). Therefore, fusion genes whose breakpoints are located in the 5' region are difficult to detect using long-reads.

We added the following sentences to the "Fusion genes" subsection in the Results (line 406-411); “We considered two explanations for the low overlap. Since most of the fusion genes missed by short-reads had very low expression levels (Supplemental Fig. S14B), many fusion genes with low expression levels were missed by the single approach. In addition, 28.9% of transcripts in long-reads lacked the 5' region (Supplemental Fig. S5 and Supplemental Fig. S14C, D). Therefore, fusion genes whose breakpoints are located in the 5' region are difficult to detect using long-reads.”. We also added a figure on the amount of data to Supplemental Information (Supplemental Fig. S14A).

*8) Figure 5D is very interesting. What do you conclude from that result? Please comment in the manuscript.*

Our reply; Thank you very much for this important comment. We used samples that used for whole-genome sequencing in our previous study. Therefore, a list of SVs is available. We classified fusion-gene to these supported by SVs (SV detected fusion-genes) and others (no SV detected fusion-genes), and compared the expression levels of them (Figure 5D).

Whole-genome sequencing can accurately identify clonal (high frequency) SVs, however, would miss sub-clonal (low frequency) SVs. Therefore, we considered that no SV detected fusion genes were generated by sub-clonal SVs. This result suggests that there are many sub-clonal fusion genes, and their expression levels are lower than clonal fusion genes. Although the functional importance of sub-clonal fusion genes is currently unknown, deeper RNA-seq would detect more of them.

We added the following sentences to the “Fusion genes” subsection in the Results (line 416-418); “This result suggests that there are many sub-clonal fusion genes, and their expression levels are lower than clonal fusion genes. Although the functional importance of sub-clonal fusion genes is currently unknown, deeper RNA-seq would detect more of them”.

*Minor comments:

1) The manuscript has many small errors in English grammar, spelling and style. I would strongly recommend sending it for copy editing before submitting it to a journal.*

Our reply; Thank you very much for this comment. The manuscript has been proofread by a native-English speaker.

*2) Neither the results section nor the methods section describing the sequencing that was performed specify whether it was done on a MinION or PromethION (or flongle). While this is implied elsewhere in the paper, it should definitely be specified in the methods at a minimum.*

Our reply; Thank you for this comment. We used a MinION for sequencing. We added the following sentences to the Method section (line 584-585); “Libraries were sequenced on a SpotON FlowCell MKⅠ(R9.4) (Oxford Nanopore) using the MinION sequencer (Oxford Nanopore)”.

*3) You also write in the introduction that your method, SPLICE, was developed for the MinION specifically. Please comment on its applicability to data generated on the PromethION and flongle Nanopore sequencers.*

Our reply; Thank you very much for this comment. We consider that our method is applicable to data from PromethION and flongle as well. We added the following sentence to the Methods section (line 597-598); “In the present study, we analyzed sequence data from MinION, but our method is applicable to data from PromethION and flongle as well.”.

*4) The volcano plot in Fig 3A is missing its dots.*

Our reply; Thank you very much for this comment. We modified the Fig. 3A.

*Reviewer #3: General comments:

Summary: In this manuscript, Kiyose et al have developed and tested a novel methodology for identifying splicing alterations, and fusions, from full-length transcript or long read sequencing data. They apply this approach to liver cancer and paired, non-cancerous liver tissue from a prior publication, and use wet-lab/experimental methods to validate their in silico findings. They conclude that their new methodology, SPLICE, outperforms one existing method, and is uniquely suitable to identifying fusion genes.*

*Major Comments:
1) Figure 1B shows a schematic of common error patterns from MinION cDNA sequencing, and the text of the manuscript describes how the authors' new approach (SPLICE), overcomes several of these, e.g. sequencing errors, artificial chimeras, and mapping errors of highly homologous genes. However, there is a fundamental disconnect between the text and the graphic in Figure 1B. This should either be revised for clarity, or an additional graphic or flowchart placed in the supplementary materials to clearly show *how* SPLICE overcomes each of these limitations.*

Our reply; We apologize for the insufficient explanation in Figure 1. We showed a detailed explanation of the data analysis procedure in Supplemental Fig. S1.

*2) Why was TALON the only alternative approach chosen for validation of SPLICE performance? There are a number of other, more advanced pipelines such as SUPPA2, and IsoformSwitchAnalyzeR. It would strengthen the manuscript, and its conclusions, to incorporate at least one of these methods as a second comparator. This is particularly true for IsoformSwitchAnalyzeR, since Kiyose et al identify a number of differentially expressed transcripts (DETs) for genes that are not differentially expressed.*

Our reply; Thank you very much for this important comment. Another reviewer also requested additional benchmarking, therefore we performed an additional performance comparison for the revised manuscript. As SUPPA2 and IsoformSwichAnalyzeR are used to analyze the annotated output GTF files, and direct comparison with SPLICE is difficult. Since IsoformSwichAnalyzeR recommends StringTie as an annotation soft, we compared using StringTie instead.

We compared the performance of SPLICE with that of four other methods (TALON, FLAIR, StringTie and Bambu) for the splicing variant detection. SPLICE identified the third highest number of transcripts after FLAIR and StringTie (Supplemental Fig. S3A). For MCF-7 , SPLICE had the highest concordance rate with IsoSeq MCF-7 transcriptome data for known transcripts and second highest for novel transcripts (Supplemental Fig. S3B).

We added the text to the "Comparison of SPLICE method with other tools" subsection of the Results (line 177-189) and the "Benchmarking" subsection of the Methods (line 644-669). We added the results to Supplemental Fig. 3.

*3) The Venn diagram in Figure 5C appears to show that conventional short read sequencing identifies 46 fusion genes that are not also detected by long read sequencing. However, this result, and its implications are never addressed in the text.*

Our reply; Thank you very much for this important comment. We apologize for the insufficient explanation. We considered two explanations for the low overlap. Since most of the fusion genes missed by short-reads had very low expression levels (Supplemental Fig. S14B), many fusion genes with low expression levels were missed by the single approach. In addition, 28.9% of transcripts in long-reads lacked the 5' region (Supplemental Fig. S5 and Supplemental Fig. S14C, D). Therefore, fusion genes whose breakpoints are located in the 5' region are difficult to detect using long-reads.

We added the following sentences to the "Fusion genes" subsection in the Results (line 406-411); “We considered two explanations for the low overlap. Since most of the fusion genes missed by short-reads had very low expression levels (Supplemental Fig. S14B), many fusion genes with low expression levels were missed by the single approach. In addition, 28.9% of transcripts in long-reads lacked the 5' region (Supplemental Fig. S5 and Supplemental Fig. S14C, D). Therefore, fusion genes whose breakpoints are located in the 5' region are difficult to detect using long-reads.”. We also added a figure on the amount of data to Supplemental Information (Supplemental Fig. S14A).

*Minor Comments:
1) On pages 20-21, the language used to describe the HBV and/or HCV postive vs negative materials is very confusing. Please clarify that by "HBV- and HCV-related tissues" you in fact mean "HBV-and HCV-infected samples."*

Our reply; We apologize for the confusing wording. We converted "HBV and HCV-related tissues" to " HBV and HCV-infected samples" in the manuscript.
