# Supplementary material for: Comprehensive analysis of full-length transcripts reveals novel splicing abnormalities and oncogenic transcripts in liver cancer
Source: PLoS Genet. 2022 Aug 4;18(8):e1010342. doi: 10.1371/journal.pgen.1010342 (PMC9380957; doi:10.1371/journal.pgen.1010342)
Supplement: Attachment — Submitted filename: Reply_to_reviewers_comments_220702.fuji2.docx [file pgen.1010342.s044.docx]

Peter McKinnon,

Section Editor: Cancer Genetics

*PLOS Genetics*

Dear Prof. Peter McKinnon,

We greatly appreciate the reviewers’ very insightful comments. To address the comments, we changed the source code of our software and modified the text.

Once again, we are profoundly appreciative of the reviewers’ comments and advice.

Sincerely,

Akihiro Fujimoto, Ph.D.

Professor

Department of Human Genetics

Graduate School of Medicine

The University of Tokyo

7-3-1 Hongo, Bunkyo-ku, Tokyo, 113-0033, Japan

E-mail: [afujimoto@m.u-tokyo.ac.jp](mailto:afujimoto@m.u-tokyo.ac.jp)

Reply to reviewers’ comments.

Reviewer #1:

1.     Regarding their reply, I only need a clarification on their rebuttal to “major point 1”: the Authors state that “If the results are inconsistent (for example, GeneA-GeneB in the reference genome and GeneA-GeneB in the transcriptome genome, or GeneA-GeneB in the reference genome and GeneA in the transcriptome genome), SPLICE considers the candidates as false positive and removes them from the analysis.”. Yet the condition “GeneA-GeneB in the reference genome and GeneA-GeneB in the transcriptome genome” looks consistent to me. This is probably a clerical error since in figure S1 the condition “GeneA-GeneB in the reference genome and GeneA-GeneB in the transcriptome genome” is used to classify the gene-fusion as consistent.

**Our reply**: Thank you for much for this comment, and we also apologize for the mistake. The correct reply is as follows. “If the results are inconsistent (for example, *GeneA-GeneB* in the reference genome and *GeneA-GeneC* in the transcriptome genome, or *GeneA-GeneB* in the reference genome and *GeneA* in the transcriptome genome), SPLICE considers the candidates as false positive and removes them from the analysis.”

2.     I also kindly ask the Authors to provide a web link to access the sequencing data deposited in the National Bioscience Database Center (NBDC). Please clarify where the dataset JGAD000635 has been deposited, is it NBCD (as stated in M&M) or NCBI as stated in the data availability declaration? Related to this, from the Authors declaration, it is unclear what data will not be made available.

**Our reply:** Thank you so much for this comment. We added the URL to the manuscript as follows (line 747); “The sequencing data have been deposited in the National Bioscience Database Center (NBDC) under accession JGAD000635 (<https://humandbs.biosciencedbc.jp/en/hum0182-v5).>”

        All sequence data used in this study are available in accordance with NBDC policy (<https://humandbs.biosciencedbc.jp/en/nbdc-policy>).

Reviewer #2:

1.     Having the code packaged in Docker certainly helps enormously. I do think that it will be difficult to maintain moving forward.

**Our reply:** Thank you so much for this comment. We built the pipeline using a Python package called Luigi (<https://github.com/spotify/luigi>) instead of running the Python code in BASH. This should improve the usability and the maintainability of the SPLICE pipeline.

2.     This is definitely good to look into. I think you should probably also address recent comments by Oxford Nanopore in talks about how the 109 and earlier sequencing chemistries had issues with mid-transcript priming at homopolymer As, which they claim to have resolved in the 110 and later chemistries. This may be the cause of the issues you observed.

**Our reply:** Thank you so much for this comment. We changed the sentence “We considered two explanations for the low overlap. Since most of the fusion genes missed by short-reads had very low expression levels (**Supplemental** **Fig. S14B**), many fusion genes with low expression levels were missed by the single approach. In addition, 28.9% of transcripts in long-reads lacked the 5' region (**Supplemental Fig. S5** and **Supplemental Fig. S14C, D**). Therefore, fusion genes whose breakpoints are located in the 5' region are difficult to detect using long-reads.” to “We considered the following three explanations for the low overlap. (1) Since most of the fusion genes missed by short-reads had very low expression levels (**Supplemental** **Fig. S14B**), many fusion genes with low expression levels were missed by the single approach. (2) In addition, 28.9% of transcripts in long-reads lacked the 5' region (**Supplemental Fig. S5** and **Supplemental Fig. S14C, D**). Therefore, fusion genes whose breakpoints are located in the 5' region are difficult to detect using long-reads. (3) Since artificially truncated cDNA can be produced by internal poly-A priming without priming to poly-A at the 3' end of the mRNA, fusion genes with breakpoints in the 3' region may also be missed.” (line 406-413)
